# Accuracy of alcohol and breast cancer risk information on Drinkaware’s website

**DOI:** 10.1111/dar.12676

**Published:** 2018-02-12

**Authors:** John Larsen, Paul Wallace, Fiona Sim, Jonathan Chick, Sarah Jarvis, Iona Lidington, Stephen Neidle, Graham Ogden, Lynn Owens

**Affiliations:** ^1^ Drinkaware—Research and Impact London UK; ^2^ University College London UK; ^3^ National Health Service England London UK; ^4^ Edinburgh Napier University Edinburgh UK; ^5^ Kingston Primary Care Trust Kingston UK; ^6^ Dental Hospital and School University of Dundee Dundee UK; ^7^ Alcohol Services/Hepatology Royal Liverpool and Broadgreen University Hospitals National Health Service Trust Liverpool UK

**Keywords:** alcohol, cancer risk, alcohol education, risk communication, public health

## Abstract

A recent paper in Drug and Alcohol Review analysed the information on cancer disseminated by 27 alcohol industry funded organisations. The independent UK alcohol education charity Drinkaware was among the organisations whose information was studied, and based on the analysis claims were made of misrepresentation of evidence about the alcohol‐related risk of cancer and alcohol industry influence. This commentary challenges the validity of these findings in respect to the evidence relating to the Drinkaware information, as the analysis is found to be misrepresenting the information by both disregarding the wider information content provided and the order and prominence with which alcohol‐related cancer risk is presented. Furthermore, it is argued that the public has a right to be provided with relevant evidence‐based information about cancer risk. It is critical that Drinkaware’s important public health function is not compromised by unjustified allegations of inaccuracy and by unwarranted attacks on its independence and integrity.

As medical and scientific advisors to and responsible for research within the independent UK alcohol education charity Drinkaware, we write in response to the paper by Professor Mark Petticrew *et al*. ‘*How alcohol industry organisations mislead the public about alcohol and cancer*’ published online in *Drug and Alcohol Review* on 7 September 2017 1. This paper reports on a study designed to analyse the information on cancer disseminated by alcohol industry funded organisations, most commonly Social Aspects Public Relations Organisations. It reportedly aimed to determine the extent to which the organisations selected for study fully and accurately communicate the scientific evidence on cancer.

The authors report the findings of a qualitative analysis of information related to alcohol and cancer risk obtained from the websites and documents of 27 organisations. The analysis is reported to have involved extraction of data independently by two authors, who coded and analysed data using documentary analysis methods. They report their method as comprising: ‘reading and understanding meanings of individual texts to identify subthemes; identification of thematic clusters of nodes; triangulation between documents and organisations; checking for reliability/validity and the use of representative examples.’

The authors claim that the analysis demonstrated that most of the organisations included in the study were disseminating misrepresentations of the evidence about the association between alcohol and cancer, and that strategies of ‘denial/omission’, ‘distortion’ and ‘distraction’ were employed. The paper concluded that the alcohol industry appears to be engaged in extensive misrepresentation of evidence about the alcohol‐related risk of cancer through its influence on the organisations studied.

Drinkaware was included among the 27 organisations considered. We address in this commentary the claims made in the paper specifically concerning Drinkaware, as we are only involved with information content provided by this organisation.

Looking at the substance of the analysis, material from Drinkaware is explicitly mentioned in relation to the ‘distraction’ strategy as an example of seeking to minimise the role of alcohol by pointing to a wide range of other risk factors, presenting alcohol as ‘just one risk among many’. The one quote from the Drinkaware website that is presented in the manuscript is the following:

*‘For example, the fact that you are female is a risk factor in developing breast cancer. We also know breast cancer is age‐related so you're more likely to develop it as you get older and that you're more prone to breast cancer if it is part of your family history. These are all factors beyond our control. We also know that risk is related to the ‘hormone environment’ that women experience during the course of early pregnancy, child birth and breastfeeding which all exert a protective effect.’*

Petticrew *et al*. claim that providing this information is misleading because it emphasises potential moderating factors ‘without acknowledging the clear independent risk of alcohol consumption’. However, all the statements made in this paragraph have long been accepted as mainstream opinion in the breast cancer medical and scientific community and there is overwhelming evidence of their correctness. As medical and scientific advisors we stand by the importance of including this information, and it is our opinion that without this paragraph we would have failed to provide women with a complete picture of the risks of alcohol and breast cancer, making us guilty of precisely the crimes of omission of which Petticrew *et al*. accuse other bodies.

Furthermore, in using only this quote the authors fail to make any reference to the comprehensive provision of information in the preceding paragraphs on the same Drinkaware webpage: ‘Alcohol and breast cancer’ (provided in Supplementary Table 2 published together with the Petticrew *et al.* paper). This webpage clearly, comprehensively and unequivocally sets out the evidence linking alcohol to the causation of breast cancer and its sources. The first section on the webpage presents the research evidence: ‘It is clear from a number of large scale studies that there is a link between alcohol consumption and cancer. Globally, one in five (21.6%) of all alcohol‐related deaths are due to cancer. Breast cancer is the most common cancer among women’. Below this, three key points are emphasised: ‘[t]here is good evidence to suggest that alcohol increases the risk of developing breast cancer’; ‘[o]f course drinking alcohol does not mean you will automatically get breast cancer, it does mean your risk of developing it will be increased’ and ‘[h]ow much you drink over your lifetime is what increases the risk’. When presenting further details of the research evidence it is highlighted that ‘[a] number of studies have found that a woman’s risk of breast cancer increases by 7–12% for every 10 g of alcohol per day’. Throughout, the relevant primary sources for these evidence claims are clearly referenced. Other webpages on the Drinkaware website address the link between alcohol and several other cancers, with which we note Petticrew *et al*. do not find fault.

Having reviewed very carefully the page singled out for criticism by Petticrew *et al*., we stand by its content and refute any misrepresentation of the clear independent risk of alcohol consumption in relation to breast cancer, either intentional or unintentional. Therefore, such criticism is, in our considered opinion, wholly unjustified and unprofessional.

Furthermore, we categorically refute the authors’ inference that information on alcohol and breast cancer provided by Drinkaware was influenced in any way by the alcohol industry. Drinkaware was established in 2007 as an independent alcohol education charity. It is run by an independent Board of Trustees, and all medical and health information provided by Drinkaware is developed with guidance from and approval of an independent Medical Advisory Panel of senior medical and scientific experts. Drinkaware is acknowledged by UK government agencies to be a major provider of evidence‐based public information on alcohol in the UK, with more than 9 million individual website visitors each year 2 and in excess of 400,000 people have downloaded the Drinkaware ‘Track and Calculate Units’ app 3.

It is vital that Drinkaware’s important public health function is not compromised by unjustified allegations of inaccuracy and by entirely unwarranted attacks on its independence and integrity. We therefore expect Petticrew *et al*. to address the inaccuracies in their paper, which we have highlighted in this commentary.
